# Combined Idiopathic Macular Hole Vitrectomy with Phacoemulsification without Face-Down Positioning

**DOI:** 10.1155/2012/571748

**Published:** 2012-02-20

**Authors:** Fumihiko Yagi, Seiji Takagi, Goji Tomita

**Affiliations:** Department of Ophthalmology, Toho University Ohashi Medical Center, 2-17-6 Ohashi, Meguro, Tokyo 153-8515, Japan

## Abstract

*Purpose*. To evaluate the outcome of combined vitrectomy with phacoemulsification without postoperative face-down positioning for idiopathic macular holes (MHs). *Design*. Retrospective, observational case series. *Participants*. Forty-two eyes of 42 patients with MH. *Methods*. We studied 42 eyes of 42 cases followed up for 6 months postoperatively. MH closure rate and preoperative and postoperative visual acuity (VA) were evaluated. *Main Outcome Measures*. MH closure rate and VA were evaluated after combined vitrectomy with phacoemulsification without postoperative face-down positioning. *Results*. Of the 42 holes, 40 (95.2%) were initially closed, and the final closure rate was 100%. Compared with preoperative VA, the mean VA was significantly improved at 1 month and the improvement was maintained for at least 6 months postoperatively. *Conclusions*. Combined vitrectomy with phacoemulsification without postoperative face-down positioning produced favorable anatomic and functional results for MH repair. Improvement in VA can be expected for up to at least 6 months postoperatively.

## 1. Introduction

Since 1991, stage 2, 3, and 4 idiopathic macular holes (MHs) have benefited from effective treatment as described by Kelly and Wendel [1]. The surgical procedure comprises pars plana vitrectomy, without cataract surgery, removal of adherent cortical vitreous, stripping of epiretinal membranes, total fluid-air exchange, and SF_6 _ gas tamponade, with strict occiput-up postoperative positioning for at least 1 week. This face-down positioning is uncomfortable, and extended periods of such positioning can also prolong immobilization, leading to ulnar nerve neuropathy [2] and pressure sores [3]. Tornambe et al. [4] first challenged the necessity of face-down positioning in MH surgery in 1997. In their study phakic eyes underwent combined cataract extraction with posterior chamber intraocular lens (IOL) insertion using 15% C_3_F_8_ gas and no internal limiting membrane (ILM) peeling, and the primary closure rate was 79%.

 We previously reported the results of a pilot study of 21 eyes of 21 patients that underwent vitrectomy with ILM removal, and SF_6_ gas tamponade without postoperative face-down positioning for MH [5]. The surgical procedure involved cataract extraction and IOL implantation in all phakic eyes (20 of 21 eyes). We present herein the results of a larger number of cases with a longer postoperative followup.

## 2. Patients and Methods

This retrospective case series included 42 eyes of 42 patients undergoing combined vitrectomy with phacoemulsification performed by a single surgeon (F. Y.), without postoperative face-down positioning. The procedures were performed at the Department of Ophthalmology, Toho University Ohashi Medical Center, from September 2006 to February 2011. Patients whose MH duration was longer than 6 months or unknown who had secondary MH, such as that due to trauma, chronic cystoid macular edema, or high myopia, and patients with clear lenses or pseudophakia were excluded from the study. After the risks and benefits of combined vitrectomy with phacoemulsification without postoperative face-down positioning had been thoroughly explained, written informed consent was obtained from all patients. The informed consent process and the corresponding consent forms were approved by the ethics review committee of Toho University Ohashi Medical Center. Institutional Review Board/Ethics Committee approval was obtained. All procedures conformed to the tenets set forth in the Declaration of Helsinki.

 MH was diagnosed by stereoscopic biomicroscopy and optical coherence tomography (OCT) and graded according to the Gass classification [6]. Of the 42 patients, 32 (76.2%) were women and 10 (23.8%) men, with mean age of 64.4 years (range, 52–79). Among the 42 eyes, 7 (16.7%) had stage 2, 27 (64.3%) stage 3, and 8 (19.0%) stage 4 MH. Mean MH size was 0.33 disc diameters (range, 0.2–0.6). MH had been present for an average of 2.5 months (range, 1–6 months). All 42 eyes were phakic.

 The surgical procedure involved cataract extraction and IOL implantation before standard 20 G or 25 G three-port vitrectomy. No posterior capsule rupture or anterior capsule tear occurred during surgery. Core vitrectomy was followed by surgical creation of a posterior hyaloid detachment in eyes with either stage 2 or 3 MH. Indocyanine green (ICG) diluted to a concentration of 0.125% was used to aid ILM peeling. The ILM was peeled beyond a radius of 1 disc diameter around the MH. Fluid-air exchange was performed, with no attempt to aspirate fluid from the MH, and the vitreous cavity was exchanged with double-filtered 20% SF_6_ gas in all cases. Patients were prohibited from assuming a face-down or face-up position until the gas had been completely absorbed.

 Preoperative data collected included age at surgery, sex, eye (left or right), visual acuity (VA) and MH stage, size, and duration. The MH status of all patients was evaluated before and at 1, 3, and 6 months after surgery by stereoscopic biomicroscopy and OCT. VA was also examined at these time points using a decimal chart, and the values were then converted to the logarithm of the minimum angle of resolution (log MAR) scale. We evaluated the longevity of 20% SF_6_ gas bubbles until confirmation of MH closure or nonclosure after surgery. The Statistical Package for the Social Sciences version 17.0 (SPSS Inc., Chicago, IL) was used for statistical analyses. The numerical data were analyzed using repeated measures analysis of variance (ANOVA). A *P* value of 0.05 or less was considered statistically significant.

## 3. Results

Patient data are shown in Table 1. Of the 42 MHs, 40 (95.2%) holes closed after the first surgery, and 2 holes did not. In these latter 2 cases, an additional 0.75 mL of 100% SF_6_ gas was injected with 3 days of face-down positioning after 20% of the SF_6_ gas used for the initial surgery had been absorbed. This resulted in closure of one MH (case 11). For the remaining eye (case 17), additional surgery was performed in which the radius of the ILM removal was enlarged to 1 disc diameter wider than that in the previous surgery. Then, the vitreous cavity was exchanged with 20% SF_6_, followed by face-down positioning. MH closure after absorption of the gas bubble was confirmed 10 days later. The final closure rate was thus 100%, and no eyes showed reopening.

 The preoperative mean VA (log MAR) of 0.65 significantly improved to 0.43 at 1 month, 0.35 at 3 months, and 0.29 at 6 months after surgery (*P* < 0.0001, repeated measures ANOVA). The mean longevity of 20% SF_6_ gas bubbles, until confirmation of MH closure or non-closure after surgery, was 7.6 days (range 4–11 days).

Iatrogenic retinal tears requiring endophotocoagulation occurred intraoperatively in 14 (33.3%) eyes. Three (7.1%) of these patients (cases 14, 26, and 33) had postoperative rhegmatogenous retinal detachment that required additional surgery to reattach the retina. One (2.4%) of these patients (case 33) had papillary capture. There were no complications attributable to ICG staining and no posterior capsular opacification.

## 4. Discussion 

Guillaubey et al. [7] compared results between a face-down position and a seated positioning after MH surgery. They speculated that the mechanism of MH repair with the patient seated is as follows. Intraocular gas tamponade has two main properties: surface tension and buoyancy. Buoyancy is related to density and surface tension is related to the viscosity of the tamponade product. The surface tension represents contact around the entire interface with the retina. The buoyant force is maximal at the apex of the bubble, depending on gravity and tamponade product depth. After MH surgery, the buoyant force is of minor significance because the most important feature of the bubble is its surface tension, which leads to closure of the hole by keeping its edge dry, independent of buoyancy. Thus, with a sufficient volume of intraocular gas, the position of the eye after intraocular tamponade should not influence surface tension around the MH. 

 We hypothesized that it does not matter whether the patient is seated or otherwise without face-down positioning, that is, that positioning strategy is not relevant because the mechanism of MH repair is the same regardless of the postoperative position of the patient. 

 In 2001, Simcock and Scalia [8] reported a 90% success rate phacovitrectomy without prone posture for stage 2 and stage 3 MH using 20% C_2_F_8_ gas and no ILM removal. In 2008, Madgula and Costen [9] reported on 31 eyes with combined phacovitrectomy for MH using 16% C_3_F_8_ gas with ILM peeling without prone posturing. Primary anatomical hole closure was achieved in 96.7% eyes. In 2010, Heath and Rahman [10] reported a 92.5% success rate of 23-gauge vitrectomy with phacoemulsification without face-down posturing, using 16% C_2_F_6_ gas with ILM peeling. 

 In the present series, surgery was performed with ILM peeling, 20% SF_6_ gas tamponade, and no face-down positioning and the anatomical success rate was 95.2% with a single operation and 100% with a single additional surgery. These findings suggest that combined vitrectomy with phacoemulsification without postoperative face-down positioning and 20% SF_6_ gas may be as effective for achieving hole closure as C_3_F_8_ gas. Patients can return earlier to their usual activities after surgery with SF_6_ gas because this gas is absorbed more quickly than C_3_F_8_ gas. 

 The methods and results of various studies of combined vitrectomy with phacoemulsification without postoperative face-down positioning are summarized in Table 2. 

Vitrectomy in phakic elderly patients results in the development of nuclear sclerotic lens opacity [11] and large long-acting gas to fill in the vitreous cavity can induce a more immediate “gas” cataract. All elderly phakic patients undergoing MH surgery will therefore develop cataracts. Combined vitrectomy with phacoemulsification has many advantages for both the patient and surgeon [12–14]. The patient does not need to return for additional surgical procedures and the surgeon does not need to perform a technically difficult operation. This is due to the lack of vitreous support which may result in unstable anterior chamber depth and variable pupil size during cataract surgery [15]. In patients with clear lenses, vitrectomy without phacoemulsification with postoperative face-down positioning for a few days was performed so that they did not develop cataracts. 

In this study, iatrogenic retinal tears occurred intraoperatively in 14 (33.3%) eyes, and 3 (7.1%) of these patients had postoperative rhegmatogenous retinal detachment. This rate appears to be higher, but Hotta et al. [16] reported that patient with peripheral retinal breaks or retinal detachment had significantly shorter duration of MH symptoms (3.2 months) than those without these complications (10.0 months). In the present series, MH had been present for an average of 2.5 months (range, 1–6 months). We plan to find between iatrogenic retinal tears or rhegmatogenous retinal detachment and duration of MH in the near future. 

Combined vitrectomy with phacoemulsification, ILM removal and SF_6_ gas tamponade followed by no face-down positioning is a potentially useful option for patients in whom a postoperative face-down positioning is difficult. In addition, postoperative care without face-down positioning is simpler and more convenient. 

 The limitations of our study include the lack of a concurrent control group and the fact that a single vitreo-retinal surgeon performed all procedures. Therefore, there are no data to compare the results between different surgical procedures or between surgeons or for evaluating the accuracy of the success rate. 

In conclusion, in 42 eyes of 42 patients, combined vitrectomy with phacoemulsification, ILM peeling, and SF_6_ gas tamponade for MH without postoperative face-down positioning achieved favorable anatomical and functional results. These surgical methods may spare patients the potentially unnecessary inconvenience of postoperative face-down positioning. 

##  Conflict of Interests

No conflicting relationship exists for any authors. The authors have no proprietary or commercial interest in any materials discussed in this paper.

## Figures and Tables

**Table 1:** Patient data.

Patient	Sex	Age	Eye	Symptom duration	Pre operative VA	Post operative VA	Hole stage	Hole size	MH status	Confirmed time
		(years)		(months)	(log MAR)	(log MAR)		(DD)		(days)
1	F	62	R	1	1	0.2	2	0.2	Closed	8
2	F	75	L	1	0.5	0.2	3	0.2	Closed	8
3	F	70	R	1	0.4	0	2	0.25	Closed	4
4	F	62	R	2	1	0.7	3	0.3	Closed	8
5	F	55	R	1	0.5	0.2	2	0.2	Closed	7
6	F	73	L	1	1	0.2	4	0.5	Closed	7
7	M	63	L	1	0.4	0	3	0.25	Closed	9
8	F	69	R	3	0.5	0.3	2	0.3	Closed	7
9	F	53	R	5	1	0.5	3	0.6	Closed	7
10	F	68	L	2	1	0.7	3	0.5	Closed	9
11	F	63	L	5	0.5	0.2	3	0.5	Open	4
12	F	58	L	1	0.5	0	2	0.2	Closed	6
13	F	64	L	2	0.3	0.5	3	0.4	Closed	5
14	F	61	R	2	0.7	0.5	3	0.5	Closed	6
15	F	65	L	2	0.3	0.4	3	0.3	Closed	5
16	F	63	R	2	0.5	0.2	4	0.25	Closed	8
17	F	64	L	2	1	0.7	4	0.5	Open	6
18	F	59	R	4	0.5	0.4	4	0.3	Closed	7
19	M	67	L	6	0.7	0.4	3	0.4	Closed	8
20	M	70	L	3	0.4	0.4	3	0.2	Closed	9
21	F	57	L	6	0.3	0	4	0.5	Closed	9
22	F	59	R	3	0.3	0.1	3	0.3	Closed	8
23	F	68	L	5	0.7	0.3	3	0.4	Closed	6
24	M	60	R	2	0.7	0.3	2	0.2	Closed	10
25	M	65	L	2	0.7	0	3	0.2	Closed	5
26	M	68	R	1	0.6	0.7	3	0.4	Closed	11
27	F	56	R	4	0.2	0	4	0.3	Closed	7
28	M	58	R	3	0.7	0.1	3	0.3	Closed	8
29	F	72	L	1	0.6	0.4	3	0.3	Closed	7
30	F	52	L	4	0.4	0.1	3	0.3	Closed	8
31	F	65	R	1	0.7	0.2	4	0.2	Closed	8
32	F	76	R	3	0.7	0.2	3	0.3	Closed	9
33	M	72	L	1	0.7	0.7	3	0.3	Closed	10
34	M	60	R	2	1	0.5	3	0.5	Closed	11
35	F	70	R	1	0.4	−0.1	3	0.2	Closed	8
36	M	66	R	2	1	0.3	3	0.2	Closed	7
37	F	62	R	2	0.5	0.1	4	0.3	Closed	8
38	F	65	L	5	1	0.4	3	0.3	Closed	6
39	F	66	R	1	1	0.3	3	0.2	Closed	8
40	F	54	L	2	0.2	0.2	3	0.2	Closed	8
41	F	79	R	1	0.7	0.2	2	0.3	Closed	10
42	F	69	R	6	1	0.4	3	0.6	Closed	9

M: male, F: female, L: left, R: right, VA: visual acuity, log MAR: logarithm of minimum angle of resolution, DD: disc diameter, MH: idiopathic macular hole, MH status: MH status after first surgery, confirmed time: time at which MH closure or non-closure was confirmed postoperatively.

**Table 2:** Methods and results of studies of combined vitrectomy with phacoemulsification without postoperative face-down positioning.

Study	Year	Gas used	ILM peeling	Hole stage (no. of eyes)	Primary (final) success rate	Followup (months)
Tornambe et al. [4]	1997	15% C_3_F_8_	No	2, 3, and 4 (33)	79% (85%)	6–40
Simcock and Scalia [8]	2001	20% C_2_F_6_	No	2 and 3 (20)	90%	3–36
Madgula and Costen [9]	2008	16% C_3_F_8_	Yes	2, 3, and 4 (31)	96.7%(100%)	10
Heath and Rahman [10]	2010	16% C_2_F_6_	Yes	3 and 4 (40)	92.5%	2–12
Present study		20% SF_6_	Yes	2, 3, and 4 (42)	95.2% (100%)	6
